# Characterizing caregivers of youth at risk for substance use and caregiver engagement in the youth legal system: a mixed methods approach

**DOI:** 10.3389/frhs.2025.1455111

**Published:** 2025-04-08

**Authors:** Annie Turner, Casey A. Pederson, Eduardo Salgado, Allyson Dir, Zachary Adams, Tamika Zapolski, Leslie Hulvershorn, Matthew C. Aalsma

**Affiliations:** ^1^ Wood College of Osteopathic Medicine, Marian University, Indianapolis, IN, United States; ^2^ Adolescent Behavioral Health Research Program, Indiana University School of Medicine, Indianapolis, IN, United States; ^3^ Department of Psychiatry, Indiana University School of Medicine, Indianapolis, IN, United States; ^4^ Department of Psychology, Indiana University Indianapolis (IUI), Indianapolis, IN, United States; ^5^ Department of Psychology, Indiana University Indianapolis, Indianapolis, IN, United States; ^6^ Department of Pediatrics, Division of Child Health Services Research Indiana University School of Medicine, Indianapolis, IN, United States

**Keywords:** caregivers, youth, legal staff, engagement, characterization, mixed-methods, substance use

## Abstract

**Background:**

Increasing caregiver and family participation is a key feature underlying many strategies to improve success among youth on community supervision. However, engaging caregivers in probation services remains a challenge for juvenile probation officers (JPOs), especially in families with significant needs. The goal of this study was to gain a better understanding of caregivers of legally involved youth at risk for substance use and their engagement with the youth legal system from a legal staff perspective.

**Methods:**

In this mixed-methods study, qualitative interviews were conducted with *n* = 15 youth legal staff from two midwestern counties. In addition, surveys were analyzed from *n* = 72 caregivers of youth with recent legal involvement who were also at risk for substance use in the two counties to characterize caregivers and provide context to the staff interviews.

**Results:**

Qualitative themes identified from the staff interviews included defining caregiver engagement, barriers to caregiver engagement (e.g., financial barriers, transportation barriers, caregiver substance use, and lack of parenting skills), and strategies to increase caregiver engagement. Quantitative data from the caregiver surveys focused on demographics and life circumstances of caregivers in the counties studied.

**Conclusions:**

Results highlight a wide variability in degree of caregiver participation with the youth legal system and legal staff's approaches to caregivers as well as significant barriers that caregivers face in their attempt to be involved in their youth's lives and legal cases. Additional work is needed to explore the caregiver perspective and identify the impact of specific caregiver characteristics on their youth and their youth's legal outcomes.

## 1 Introduction

Close to 2 million youth entering the youth legal system are placed on community supervision (e.g., probation) as opposed to more restrictive options that remove youth from their home (e.g., detention, correctional facility) (1, 2). On probation, youth complete a series of requirements under the supervision of a juvenile probation officer (JPO) while remaining in the community. These requirements may include restitution and community service, mandatory participation in meetings with their probation officer, adherence to school attendance and assignments, random searches, abstinence from drug use and drug testing, and/or use of location monitoring devices (3). However, recent census estimates suggest that up to 14% of youth on probation (i.e., approximately 280,000) do not complete their probation requirements (4) resulting in adverse consequences such as lengthened probation times, escalation of their case, or formal detention (5). In fact, some studies have found the census estimate of 14% to be low and estimate up to 52% of youth on probation do not complete probation requirements, resulting in probation violations such as positive drug tests, school adherence failures, and new arrests (6). Continued involvement in the legal system through lack of probation completion and probation violations is linked to adverse lifelong consequences such as increased recidivism, delayed psychosocial maturity, and delayed achievement of developmental adolescent milestones (e.g., timely school completion and workforce eligibility) (5). Accordingly, it is important to understand ways to maximize youths' successful completion of their probation requirements.

Increasing caregiver and family participation is a key feature underlying many strategies to increase success among youth on community supervision (7–9). Historically, caregivers were blamed for their child's engagement in delinquent behavior (10) and the youth legal system was designed under the assumption that it was necessary for the court to serve as the youth's acting caregiver under the doctrine *parens patriae*. *Parens patriae* indicates that a legal authority may step in as a caregiver for youth when necessary. While *parens patriae* is still a guiding principle for the youth legal system, efforts have recognized caregivers as important stakeholders in youth rehabilitation (11). Caregiver involvement (i.e., taking an active role in their child's life including their interactions with the youth legal system) continues to be ranked among the most important issues for youth legal systems and research programs (12, 13), and greater caregiver involvement has been linked to positive outcomes among legally involved youth including adjustment to detention (14), successful reentry (15), and reducing recidivism (16). Furthermore, family-based interventions have been shown to reduce recidivism (17, 18) and are more effective than individual-based interventions in reducing disruptive behavior (19).

Although caregiver involvement is a key predictor of youth outcomes, engaging caregivers in probation services remains a challenge for JPOs, especially in families with significant needs (20). Caregiver involvement is lower amongst legally involved youth than in the general population (21) which makes assigning probation requirements difficult as caregiver support is often necessary to successfully meet these requirements. For example, certain probation requirements require caregivers to assist with transportation as well as being present for emotional support and continued monitoring. Though these terms are imposed upon the youth, it becomes a family-level effort to meet these requirements. Caregivers' capacity to engage fully in probation requirements—and to be involved in their children's lives more generally—may be influenced by family demographics including socioeconomic status and household size, family cohesiveness and conflict, presence of life stressors, presence of substance use or criminal activities in the family, and presence of resilience factors, such as hope and life satisfaction. In many cases, the burden falls upon caregivers to help their youth successfully complete probation requirements. With a nuanced approach (i.e., identify transportation needs, have flexible hours for probation visits, aid in connection to services, etc.), court and probation staff may be able to increase caregiver engagement and improve youth outcomes. Therefore, it is important to describe the needs of caregivers with youth involved in the legal system so interventions can be tailored to meet their needs. Perspectives from legal staff surrounding caregivers of legally involved youth can give insight into the unique qualities of this population as well as the circumstances of their lives. Legal staff can provide insight on current levels of caregiver engagement, legal practices that include caregivers, and strategies to increase caregiver involvement. Previous studies have sought to identify the ideal characteristics of caregivers from a legal staff perspective (22) and to generate a framework for understanding caregiver involvement in the youth legal system (7). Below, we briefly review what is known about the importance of these factors.

A number of demographic variables may influence caregiver engagement with the youth legal system. For example, it is well known that racial and ethnic disparities exist in various stages of legal processes such that youth of color are more likely to be arrested and penetrate deeper into the youth legal system than White youth (23, 24). Researchers have attributed these differences to a combination of environmental and structural risk factors, many of which may be relevant to caregivers of legally involved youth. One environmental risk factor tied to higher involvement with the legal system may be living in an economically disadvantaged and unstable community (23–25). For caregivers with limited finances or lower socioeconomic status, living in such communities may be the only affordable option, which may have a number of unintended consequences that place youth at a higher risk of delinquency such as access to only lower performing or low resourced academic institutions (26), greater exposure to delinquent peers, interpersonal violence, gang activity (27, 28), and limited access to prevention and treatment resources (29). In fact, some studies have shown that these communities may overlap with jurisdictions that tend to have harsher law enforcement or judges compared to jurisdictions with primarily White residents (29–31). As several studies have demonstrated a relationship between low socioeconomic status and non-White race and ethnicity in the United States (32), caregivers of color may not be equipped with sufficient resources to support their children and have limited means to overcome these barriers. Families with legally involved youth tend to have a higher prevalence of family risk factors such as single caregiver households, incarcerated caregivers, higher mortality rates of family members, and higher number of family members in a single household, suggesting that caregivers may be particularly stretched thin across various domains (23, 25, 28, 33, 34), limiting their availability for engagement.

Families with legally involved youth experience higher levels of familial conflict than other families (35, 36). One possible contributing factor to this relationship may be the higher prevalence of physical abuse via harsh or punitive discipline in families with legally involved youth. There is evidence that harsh or inconsistent discipline, coercion, and/or cold or rejecting parenting styles predict later juvenile delinquency in families who utilize these techniques (37). As previously discussed, these practices may stem from caregiver inability to consistently monitor their children and/or limited skill development in alternative parenting strategies, highlighting a possible area of need for caregivers. Regardless, use of these practices can build animosity within all levels of the family unit (37). In fact, sibling conflict alone has also been shown to be predictive of antisocial behavior in these families (38). When considering that these families may have larger households (39), it becomes likely that family conflict will be prevalent and may contribute to further delinquency and/or challenges with assisting youth to meet probation requirements.

Additionally, caregivers may be struggling with their own needs going unmet, particularly as they relate to difficulties with substance use. The literature suggests that caregiver challenges with substance use, and mental health strongly predict subsequent youth substance use (40). Possible mechanisms of action for this effect have been linked to implicit and explicit pro-drug and permissive attitudes in the family (41), increased child maltreatment and subsequent use of substances to cope (40), and increased access to substances (42). Indeed, caregiver substance misuse in families with legally involved youth may be highly prevalent (40), highlighting an unmet need with potential for harmful downstream consequences for their children.

Finally, it is important to assess family-level strengths as protective factors may buffer against the harmful consequences previously mentioned. Strength-based family services increase resilience among youth and their families (43) and, when recognized by legal staff, these strengths may highlight positive ways for caregivers to engage with their youth and the legal system. Two constructs that may be important to assess in caregivers of legally involved youth are hope and life satisfaction. Hope, defined as a cognitive process that helps people to have a positive expectation to reach desired goals and to perceive that goals can be met (44), may relate to the degree to which the family engages with the legal system given the uncertainty surrounding youth legal involvement. High levels of hope have been linked to higher levels of psychological resilience, higher motivation, and fewer mental health symptoms; these critical protective factors may be necessary for engagement with adversities (44) which is particularly relevant for caregivers of legally involved youth. Though some research has been done linking hope in legally involved youth to positive outcomes (45), little work has been done to understand the role that hope plays for caregivers of legally involved youth. Given the involvement of caregivers in their youth's lives as well as the high demands imposed on caregivers by probation requirements, it is important to understand whether caregivers remain hopeful in these situations. As youth look to their caregivers for role models, increased caregiver hope may also increase youth hope. Another construct, caregiver life satisfaction, may have downstream effects on their youth's life satisfaction. Given the previously reviewed literature on typical living conditions for families with legally involved youth, it may fall to caregivers to model the degree of satisfaction with their lives to their children. In fact, studies have shown that youth's global life satisfaction may relate to family life (46) and caregiver life satisfaction (47).

Taken together, this brief review highlights the tumultuous circumstances experienced by many families with legally involved youth at risk for substance use. This study aims to characterize caregivers of legally involved youth at risk for substance use to better understand the obstacles they face as well as their strengths that can be leveraged to best help their youth navigate the legal system. This study includes open-ended data from interviews with probation officers and legal staff at participating sites to obtain staff's perspective of caregivers and identify simple methods that are currently being used to increase caregiver engagement. This study also includes survey data from caregivers of youth with legal involvement at these sites to provide context to the staff interviews and corroborate staff perceptions. We believe that this study will provide us with a better understanding of caregivers of legally involved youth at risk for substance use and their engagement with the youth legal system. With a better understanding of caregivers' unique situations, JPOs and legal staff will be able to modify interventions and policies to target specific needs and maximize caregiver participation with the youth legal system.

## 2 Methods

### 2.1 Research design overview

Data for this analysis were collected as a part of a hybrid type 1 clinical-effectiveness-implementation trial working with youth legal systems and community mental health centers (CMHCs) to identify legally involved youth in need of substance use treatment and connect them with appropriate behavioral health care. Components of the trial included introduction of rapid substance use screening for youth encountering the legal system, stratification of youth according to risk, and training of CMHCs to utilize evidence­-based substance use treatment specific to youth's risk level. More information about the parent study can be found in the published study protocol (48). For this study we use a convergent-parallel mixed methods design with an inductive-deductive thematic qualitative approach and descriptive quantitative design.

### 2.2 Setting

This study took place in two youth legal systems in a midwestern state. Two counties were included to achieve an adequate sample size. One county was rural with a metropolitan area of fewer than 40,000 people, while the other was a suburban county with a metropolitan area of fewer than 250,000 people. The population in the rural county was 88% white, non-Hispanic and the population of the suburban county was 75% white, non-Hispanic. The rural county legal system employed 6 staff who had contact with adolescents, and the suburban county legal system employed 20 staff who had contact with adolescents.

### 2.3 Participants

#### 2.3.1 Quantitative

In addition to interviews with legal staff, survey data was analyzed from 72 caregiver surveys collected as a part of the larger parent study (i.e., 10 caregiver surveys from the rural county and 62 caregiver surveys from the suburban county). Caregivers were recruited for the parent study if they had a youth who had recent involvement with the youth legal system (i.e., arrested in the last year) and was identified as being at risk for problematic substance use as defined by scoring 1 or greater on the CRAFFT questionnaire (49). “Caregivers” were defined as anyone who was currently serving as a primary caregiver for the youth. The caregivers recruited for this study were mostly biological parents but also included adoptive parents, grandparents, other relatives, and non-relative guardians. The CRAFFT consists of six yes or no questions regarding substance use (e.g., “Do you ever use alcohol or drugs to relax, feel better about yourself or fit in?”, “Do you ever forget things you did while using alcohol or drugs?”) as well as three questions regarding frequency of substance use in the past year with higher scores (0–6) indicating greater risk for a substance use disorder (50). The CRAFFT has been shown to be clinically effective for identifying substance use risk level among youth (51) and validated internationally for use in youth legal systems (52). To recruit the caregiver-youth dyads, a legal administrator from each location provided contact information to the research team for the caregivers of all youth who scored 1 or greater on the CRAFFT questionnaire at intake over the span of 2 years and 3 months. A total of 380 caregiver-youth dyads were referred by legal administrators for recruitment. To be eligible to participate, youth had to be between the ages of 14 and 17 and both youth and their caregivers had to be proficient in English. Youth were excluded from participating if they were currently detained or classified as wards of the state.

Research assistants contacted the dyads and asked if they were interested in participating in the parent study. Assistants made attempts to contact the dyad until the caregiver or youth declined to participate or until they were unable to be reached on three consecutive attempts. If dyads were interested in participating, the research assistant scheduled a time to meet at their home or at a public location where they obtained written informed consent and administered the surveys on an iPad. Participants were verbally instructed to interpret the word “parent” in survey measures as referring to the primary caregiver participating in the study and interpret the word “child” in survey measures as referring to the youth that was participating with them in the study. During the COVID-19 pandemic, informed consent was provided over the phone with a digital informed consent form and online survey collection as approved by the institution's IRB. Participants were informed that their participation was completely voluntary, that participation or non-participation would not be disclosed to the youth legal system, and that all survey answers were confidential. Surveys took approximately 1 h to complete using Qualtrics survey software, and the youth and caregiver were each compensated up to $100 for their time. The amount of compensation was increased throughout the duration of the study to improve participant recruitment and retention. For this analysis, only data from the caregiver's survey responses were used due to the focus on caregiver engagement.

#### 2.3.2 Qualitative

We analyzed 26 interviews conducted with youth legal staff throughout the duration of the parent study. We report the methods of this analysis following the COREQ guidelines for reporting qualitative research (53). Staff roles were JPOs, judges, and intake staff. Perspectives from prosecution and defense lawyers were not included because many youth involved with the legal system do not undergo formal sentencing and thus do not obtain legal counsel. The interviews were conducted by the eighth author and a doctoral student. The eighth author had collaborated previously with the youth legal staff on previous projects related to criminal justice reform while the doctoral student had no prior interactions with them. Legal staff were contacted at random by a graduate research assistant from a list of staff familiar with the project which administrators from each site had provided. All staff members who were contacted agreed to participate in interviews. Interviews were conducted over the phone and lasted about 45 min. Staff were not compensated for participating in the interviews as they were state employees and unable to accept payment for their time. Interviews centered around current practices and implementation of the project. Prior to the interview, staff were told that researchers were primarily interested in their perspectives on the substance use treatment services currently available to adolescents in their communities and efforts made within the parent study to connect legally involved youth to community-based substance use treatment.

### 2.4 Measures

#### 2.4.1 Qualitative interview guide

The research team developed a semi-structured interview guide focused on staff member's perspectives on substance use treatment and intervention implementation. Topics included substance use treatment availability, current processes of referral for substance use treatment, and suggestions for improvement in the collaboration between legal staff and their local community mental health center (CMHC). Participants were encouraged to expand on their answers and interviewers asked probing questions when necessary. The research team met early in data collection to refine the interview guide and ensure that quality data was being collected. The team continued to meet throughout the collection process to discuss themes and identify when data saturation had been reached. Although parenting was not the focus of these interviews, caregivers were often mentioned in relation to the core constructs being studied. For the current study, we focused our analysis on themes relating to caregiver engagement, the impact of home life and family dynamics, staff opinions of caregivers and the caregiver role, and the caregiver-child relationship. Themes from these interviews not related to caregivers are reported elsewhere (54, 55).

#### 2.4.2 Survey measures

##### 2.4.2.1 Demographics

Caregivers reported basic demographic information, including income, age, gender, race, language, relationship status, and household composition.

##### 2.4.2.2 Family affluence

Family affluence was measured using the Family Affluence Scale, a four-item measure developed in the WHO Health Behaviour in School-aged Children Study (56) to measure family wealth. The scale asks questions regarding computer and car ownership, if the child has a room to themselves, and if the family has been on vacation in the last year. Participants can respond “yes”, or “no” as well as “yes-one” and “yes-two or more” to relevant questions. Responses were used at the item level for analysis.

##### 2.4.2.3 Caregiver hope

Caregiver hope for their lives was measured using the Hope Scale (HS) (57), a 3-item measure on a 5-point Likert scale (1 = Not at all like me, 5 = Exactly like me). The scale asks how much each statement (e.g., “I am excited about my future”) describes the person. A mean score was calculated, with a higher score indicating greater hope. Analysis supports good internal consistency in this sample (*α* = 0.83).

##### 2.4.2.4 Caregiver life satisfaction

Caregiver satisfaction with their lives was measured using the Life Satisfaction Scale (LSS) (58), a 3-item (e.g., “I am happy with my life”) measure on a 5-point Likert scale (1 = Disagree Strongly, 5 = Agree Strongly). A mean score was calculated with higher mean scores indicating a higher life satisfaction. Analysis supports acceptable internal consistency in this sample (*α* = 0.74).

##### 2.4.2.5 Caregiver alcohol use

Alcohol use was measured using the Alcohol Use Disorders Identification Test—Concise (AUDIT-C) (59), a 3-item measure with each item having its own 5-point Likert scale evaluating how often caregivers have a drink (0 = Never, 4 = 4 + times per week), how many standard drinks caregivers have in a day (0 = 1–2, 4 = 10+), and how often caregivers have six or more drinks on one occasion (0 = never, 4 = daily or almost daily). Scores were summed consistent with scoring conventions, with higher scores indicating a higher likelihood that the individual's drinking is impacting their health and safety. Scores greater than 3 are positive for likely alcohol use disorder (AUD) in women and scores greater than 4 are positive for likely AUD in men. Analysis supports acceptable internal consistency (*α* = 0.73) in this sample.

##### 2.4.2.6 Caregiver drug use

Drug use was measured using the Drug Abuse Screening Test (DAST) (60), a 10-item dichotomous measure (0 = No, 1 = Yes) regarding substance use other than alcohol in the past year. The first question of the measure asks if caregivers have used drugs other than those required for medical reasons in the last year. The remaining questions ask about the extent of caregiver's drug use with questions such as “Do you abuse more than one drug at a time?”, “Does your spouse ever complained about your involvement with drugs?”, “Have you neglected your family because of your use of drugs?”, and “Have you ever experienced withdrawal symptoms when you stopped taking drugs?” Scores are summed with higher scores indicating a greater severity of drug use.

##### 2.4.2.7 Life stressors

Life stressors were measured using the Changes and Adjustment Scale (CAS) (61), an 18-item dichotomous measure (0 = No, 1 = Yes). The CAS asks caregivers to identify life stressors that have occurred in the last year including potentially positive (e.g., “your child experienced the birth of a sibling”, “you remarried or reconciled with your child's other parent/caregiver”, “you or your child lived in a home undergoing significant repairs or remodeling”) and negative stressors (e.g., “your child was separated from you or another parent/caregiver”, “your child had an accident or injury”). The measure includes stressors directly impacting the caregiver as well as those impacting other family members and covers multiple categories of stress including financial (e.g., “you had problems at work”), legal (e.g., “You experienced legal problems”), family conflict (e.g., “Your extended family experienced conflict or other problems”), medical (e.g., “Your child was frequently or severely ill”), upheaval (“You or your child moved or relocated”), and loss (e.g., “You experienced the death of a family member”). Scores are summed with a higher score indicating a greater number of life stressors.

##### 2.4.2.8 Caregiver monitoring

Caregiver monitoring of their youth was measured using the Parental Monitoring Scale (PMS) (62), an 8-item measure on 5-point Likert scale (1 = Never, 5 = Very Often). The measure asks caregivers to rate how often they engage in specific monitoring activities (e.g., “I know where my child is after school”, “If my child is going to be home late, they are expected to call me to let me know”). Scores are summed with higher scores suggesting a higher level of monitoring. Two questions were added to the measure regarding monitoring of cell phone use and social media use. Analysis supports good internal consistency in this sample for both the original (*α* = 0.82) and the expanded measure (*α* = 0.81).

##### 2.4.2.9 Caregiver support

Caregiver support for their youth was measured using the Parental Support Scale (PSS) (63), a 10-item measure on a 5 point Likert scale (0 = Strongly Disagree, 5 = Strongly agree). The measure includes five statements describing supportive behavior (e.g., “I give my child the right amount of affection”, “I often ask my child what they are doing in school”) and five statements describing unsupportive behavior (e.g., “I sometimes put my child down in front of other people”, “I wish my child were a different type of person”) and asks caregivers to rate how much they agree with each statement. The five unsupportive statements were reverse scored. Scores were then summed with higher scores indicating a higher level of support. Analysis supports acceptable internal consistency in this sample (*α* = 0.77).

##### 2.4.2.10 Family conflict

Family conflict was measured using the family checkup measure, a 4-item measure on an 8-point Likert scale (1 = Never, 8 = Always). The scale asks how many times events such as arguing, hitting, or getting one's way by being angry occurred in the last month (e.g., “we argued”, “we got angry with each other”). Higher mean scores indicate greater family conflict. Analysis supports acceptable internal consistency in this sample (*α* = 0.74).

#### 2.4.3 Data analysis

##### 2.4.3.1 Quantitative data analysis

Descriptive statistics including percentages, means, medians, standard deviations, and correlations were calculated for all measures using SPSS software. Because data from two separate counties was used in this study, data was initially analyzed for each county both separately and together. No discrepancies between counties were identified so analysis proceeded with the combined data.

##### 2.4.3.2 Qualitative data analysis

For the purpose of this study, we conducted a secondary qualitative inductive-deductive thematic analysis (64) to understand legal staff perspectives on caregiver engagement in the youth legal system. Qualitative interview files were uploaded to Rev.com for transcription and subsequently de-identified. Transcripts were coded in Atlas.TI and Nvivo, qualitative analytic software programs for transcript coding and analysis. Codes included a combination of *a priori* codes based on the interview guides and study questions and codes based on themes that emerged throughout the coding process. Examples of initial codes include defining caregiver engagement, positive caregiver engagement, lack of caregiver engagement, and suggestions for improvement. Transcripts were coded by a research assistant and then reviewed by a faculty member No discrepancies in the initial reviewer's coding were identified by the faculty member. After the coding was completed, four members of the research team discussed the codes to condense similar codes and identify emerging themes. All codes were discussed until a consensus was reached. See Tables 1–3 for examples of codes with their corresponding quotations. Additionally, sociodemographic data was analyzed for caregivers of legally involved youth in both counties where legal staff were interviewed to provide context for staff observations and authenticate legal staff perspectives with descriptive statistics calculated for each measure. The quantitative data is presented first to briefly characterize caregivers followed by qualitative themes from legal staff to provide a comprehensive view of caregiver engagement in the youth legal system. Legal staff's discussion surrounding caregivers and caregiver involvement focused on three main categories: current opinions on caregiver engagement (Table 1), barriers to caregiver engagement (Table 2), and strategies for improving caregiver engagement (Table 3). These categories are discussed in more detail following the quantitative data.

**Table 1:** Staff perception of caregiver engagemen*t.*

Defining Caregiver Engagement	Staff Quotations
Shared decision making	*“For me, parent engagement means parents that are informed, parents that are given the opportunity to participate in the decision-making, the outcomes, the consequences that there were along the way, and that has a voice, and anything and everything that's happening with their kid.”*
Attending appointments	*“I would say good parent engagement looks like being there to support their child, attending appointments with their child, in the right setting. I realize that some appointments need to be one on one with the provider and the youth, but I think getting family involvement just adds more support to the treatment system itself.”*
Involved in all aspects of child’s life	*“the engagement piece really speaks to the parents' involvement with school, social, emotional, behavioral, all the aspects of parenting that we all expect for traditional parents or nontraditional parents.”*
Following up at home	*“parent engagement would be that the parents are engaged, that the parents are taking them to their appointments, the parents are hearing the information from all treatment providers, including the probation officer. The parents are seeking out questions from the service providers like, ‘What can we be doing at home to follow up on this stuff?’”*
Impact of Caregiver Engagement	Staff Quotations
Lasting change at home	*“To change the cycle, I think that we need the parent engagement. 'Cause we're not gonna change the cycle with just the kid. We're not gonna work with a client for six months and send them through all this treatment and think that we're gonna have life-changing behaviors. We have to make those changes with both the generations that are in the home together in order to really start making a difference in that cycle.”*
Accountability	*“One way or the other [parental engagement] always helps them [juveniles]. Sometimes the helps them 'cause then they have that daily voice in their ear reiterating what probations telling them and the police are telling them. IT's just whole comprehensive trot towards the juvenile to help encourage those changes. And even if the juvenile doesn't make those changes, as long as we don't have the parents enabling, the juvenile at least gets the clear, consistent message that these types of behaviors are gonna have consequences. The police don't need to catch you, your parents are gonna report you, your teachers are gonna report you. And now everybodys talking to each other and whatever you decide to do, you'll have to answer for. So no matter what parents, they will definitely have a good impact on the juvenile regardless of ultimately the path they choose for themselves.”*
Completing requirements	*“Overall I think parent engagement is huge, because I've had kids with parents who were completely unengaged with us down here, and I've had some that are overly engaged with us. I feel like it makes a huge difference on whether or not they're going to be successful, because if you don't have that parent pushing their child to go and get their community service done, to do their programming, stuff like that, I see that often result in them going up to court, because they're not getting their requirements done in time and not doing what they need to do. So I think parent involvement is very important…And then them getting involved and knowing what's going on so they can report any concerns to me so those can be addressed sooner rather than later as well. So like I said, I think it's extremely important. It can make a big difference on how successful the juvenile is.”* *“Sometimes we have parents who are onboard cooperative, supportive, encouraging…And then there are some that are absolutely not invested and we don't do a great job maybe including those ones [unengaged parents] that we could get more support from, and to getting the ones that aren't exactly invested, more invested. I don't know how we do that, but I would love to see that happen. I think that was would certainly improve attendance and compliance and completion of services.”* *“I feel like having parents engaged definitely helps the juveniles to get all their stuff done quicker. To help them through that process, help them get, make sure that they're getting everything completed.”*
Attitude	*“I do think it [parental engagement] is important, because I think it just makes the kid take it more serious… Kids watch their parents and I feel like the parents that come in and act like it's a joke, why would their kid take any differently?”*
Encouragement	*“I think having the parents involved in the process…can be encouraging to the kids so they see that their parents care and they want to help them, kind of help them through this process… the whole process can just be a lot. So having that extra support from the parents I think definitely helps the kid to be more, helps them to be successful.”*
Current Caregiver Engagement	Staff Quotations
Lack of caregiver engagement	*“There are some parents who don't even remember office visits, don't even help their kids with that aspect, don't help their kids get to services. They just expect them to take the bus, or do things on their own. Some parents don't show up to office visits.”* *“Unfortunately, the majority of what we see is the kid gets themselves to all their appointments, and we see the parents at court once every three months.”*
Variety of caregiver responses	*“it just seems like we either have parents who are gonna be extremely engaged and helping them. You know what I mean? Who want to help them. And then we kind of have the opposite side of the spectrum where we have somebody, where we have parents who are just like, well this is on them. They were the ones that got themselves in trouble. They need to do all this.”* *“ you've got kind of camps on both ends [of engagement], and then you've got the middle, where depending on how you approach them coming in, you can either turn them off and them end up saying ‘I don't want to cooperate. We're not doing this. We're going to get an attorney’, or you can kind of buy them over, you know what? This isn’t going to be so bad. I'll have a say in how this works, and I'll have some input.”* “*Some parents were definitely involved and supportive of their child. Others were not so much, so it varied.”*
Positive caregiver engagement	*“Oh the majority of them [parents] are [positively engaged]. Especially when you get to the end of the whole thing. They may not start out that way, but usually you can win them over, if you start saying things like ‘let's look at how your kid's been at home, there's no improvements that your child could make?’ And parents are like ‘oh no, they could be doing this or that’, then ‘okay let's start there,’ usually can win them over. I'd say most parents are pretty aware of what it is their kids are and aren't doing, they just feel the need to defend them in the moment from the big bad legal system. They'll figure you the systems really on their side, we usually to see much better results.”*

**Table 2:** Staff perceptions of barriers to caregiver engagement.

Themes	Staff Quotations
Cost and Scheduling	*“Parents usually the first thing they want to know is ‘how much is it going to cost me? Am I going to have to take off work.’”* *“the parents say, ‘We'd have to move to pay for all this stuff. We [have] bills. We can't just quit our lives to take our kids to all these appointments.’ It kinda puts them in a position where it's difficult.”* *“I've had parents actually say this to me, they feel like they're the ones getting punished, because depending on what all that kid has going on, taking them several places can be a lot.”* *“I've got a lot of kids whose parents work second shift, and so that can be a deal breaker sometimes.”*
Outpatient Transportation	*“Transportation is a huge barrier around here. We're a rural community, and [local CMHC] is only in [town name], so for someone, a kid in [nearby town], that's 30 minutes for them to get in, and some parents can't do that.”* *“There is no in-patient treatment, essentially for kids at all. Whatever outpatient treatment there is, it's not always necessarily accessible. Because kids are relying on their parents and sometimes we don't have very reliable parents. And so that's a problem, transportation can be a problem.”*
Inpatient treatment distance	*“It's something we talk about more so on the deep ends, so the kids that we send to residential or remove from the home, about that parental engagement, because they're not there to engage with the child, especially in the residential setting, so how are parents going to participate in treatment? How are parents going to visit?”* *“We don't really have like an acute facility here for kids that are suicidal. I mean they have to go to [nearest city], or somewhere else. I mean I think our mental health services are lacking. Then, and then most kids who do substance treatment, like if they do it in, like if they do a residential, also have to travel pretty far away, with makes it difficult for a parent to be able to go and participate and see them.”*
Shame	*“we have some parents come in here who I think they feel, at least at the beginning, especially like at an intake appointment, feel like we're just looking at them like man, you're a terrible parent. So sometimes that's a big thing… We do have a lot of parents who feel that's, they're definitely showing their attitude when they first get here. I think it's because they think we're thinking they're just bad parents, so I think that is a big one.”*
Substance use	*“some of the parents use with their kids. Those are the ones, and a lot of those come from impoverished towns. I would say those families, they don't see the difference [between experimentation and problematic substance use], or they don't recognize it.”* *“there are families we see where the parents are not drug users and the children have become drug users in some way. And that is a very different dynamic than the families where the parents are drug users and the kids are drug users. And so their approach to that has been no different. I think you've got to approach those situations in a different way. I don't know that you can treat those circumstances the same and expect to get great outcomes by just treating the children.”*
Lack of ownership	*“you've got the parents who say ‘I don't understand why I've got to take my kid to counseling. I don't understand why I've got to be here or I've got to take off work. I didn’t get in trouble.’ They don't take ownership in it being a family issue. They see it as just the kid's issue and not a family issue.”*
Lack of perceived need	*“I think there's always a bit of poo pooing on behalf of, oh, everybody…peers, adults, parents, law enforcement in particular, that the kid out there smoking dope's not that big of a problem. The kid out there farting around with certain drugs is not that big of a problem. ‘They're kids. This is what kids do. Doesn't mean they have a problem. Doesn't mean they will have a problem.’”* *“Quite often [parents] are in denial or they're minimizing how much of a problem it is. They're also very defensive about their child's drug use. ‘It's not as bad, they just got caught,’ those kinds of… ‘Their friends are doing it, and they didn't get in trouble so why is my kid in trouble?’"*
Overlapping roles	*“we have kids who come in who get arrested and as soon as they get home, their parents are punishing them, giving them consequences, knowing ‘yeah, he could still get put on probation, but he's still my kid,’ and then we have other parents who are like ‘we wanted to see what you guys did first.’”*
Lack of parenting skills	*“the parents that are frustrated with kids that may be a bit more system involved see it as ‘I can't control him. He knows better. She knows better. I taught him better than that,’ so maybe finding ways to share in that responsibility or ownership of that, but also understanding that certain parents have done the best that they can do, and the child's needs maybe exceed the ability… I think we see that based upon the large number of school arrests that we get, which are largely disobedience, not following orders, not following directives, school discipline issues turned delinquency issues, which to me speaks to a serious skill gap that is being transferred to the kids or being utilized by the kids.”* *“A client right now, him and his mom, or he and his mom, I know that they care about each other, but they just don't know how to keep situations from just blowing up. They're just not good at, everything turns into an argument.”*
Uninformed	*“I think that the reason kids and families struggle meeting the requirements is that they don't always understand the purpose of them.Then people feel like, ‘well, I'm just getting all these requirements and it's just really cookie cutter. Everybody has to do everything…’ No one's asking questions, or no one's really talking about why is that specific requirement or referral necessary.”* *“I think we miss that informed piece at the beginning, where parents aren't informed that they do have a voice. And so, we'll ask questions to engage them, but unless you're empowered and believe that your voice can and will be heard, even if it's not agreed with, I think that's an engagement piece that we lack.”*

**Table 3:** Staff strategies for caregiver engagement.

Themes	Staff Quotations
Respect for caregiver’s schedule	*“Friday afternoons are not ideal on my schedule because that’s when I’m trying to finish last minute work, but I’ve got a kid whose mom works every day but Fridays so we always schedule on Fridays so I think she values the fact that we value her time and another thing is always being on time. I let the parents know we will always try to see you on time, so it's not going to be like the doctor's office where you show up for a 3:00 o’clock and you're not seen until 4:00. We always try to be respectful of their time, and basically just letting the parent know that this is an effort to try to make things better at your house, and in your home with your child. Usually if you can sell it that way, they're a lot more cooperative.”*
Frequent communication	*“There are some appointments that we require the parents to attend, and usually what I tell the parents is once we get the child on probation, we would like to have your input. We want you to come. If for some reason you can't, if there is another adult that can bring them, that's helpful, so that we can kind of find out from you what's going on, but if you can't be here and your kid has to walk over after school, call me and let me know how things are going so we can kind of maybe check in over the phone and try to keep them engaged in that, because they'll very quickly fall off and you'll see a kid start showing up once a month and the parents are just kind of out of it. And so sometimes we have to really kind of work to keep the parent involved in those conversations”* *“I try to have the parents come to the office with their kids, because I think it's important that everybody's on the same page. But if worse comes to worst and they can't make it, I'm definitely calling and talking to them and making sure that what the juvenile's reporting is the same as how they feel. Making them aware of failed drug screens, and making them aware of problems at school that they might not know about. So I try my best to make sure that they're involved and that they're aware of all the problems or concerns that are coming up. Then they're involved from beginning to end as much as I can get them involved.”*
Education	*“I at least send them [parents] out with at least verbal tools, verbal praise and maybe suggestions…some ideas of how to engage with their child.”*
Family oriented programs	*"We do have a few programs that actually include the parents. Those obviously are pretty engaging and actually allow the parents who might not be receptive to kind of being a part of it actually enjoy doing those, but there's only one or two that we have like that.”* *“We've put a little bit into practice, and the feedback from parents has been really powerful…Before an emergency decision hearing, that has resulted in out-of-home placement, either from secure detention or emergency shelter care. We do what's called a facts panel, which means finding alternatives for safety and treatment. And so, we bring parents, their supports in. We've had pastors. We've had church members, anybody that they want to come in, and they come to the table, and it's the prosecutor, public defender, Department of Child Services, probation, the intake officer, and then a service provider, and the parents and their support… it's very brief. It's 15 minutes, but man, we get a lot done. It's, ‘Here's everybody who's going to be involved with your case right now. Here's what we're going to try to decide. Here's what we're going to try to determine what we can recommend to the judge. And here's all the questions that we have for you. What questions do you have for us?’ And so, it's really making the parent part of the team in that moment to figure out what the recommendation at that emergency detention hearing's going to be. Also, we do a little exit survey at the end, and parents are like, ‘He's been arrested many times before, but that is the first time I felt heard. That is the first time I understood what was going to happen in a court hearing.’ So, I think just finding ways to slow down the process and bring other people in with the parents that are connected to the youth legal system but also have their support as well, and to the mutual parties, so that it's not so overwhelming and intimidating. The more we can do that with parents to build those relationships and that trust and that empowering, I think it's really important…it's so powerful for the parents … Most of the time, parents go into that court hearing, and you can't even comprehend what's happening because it goes so fast. And so, this is just a way to say, ‘This is what's happening. This is what the plan is. That is what the concerns are. What can we calculate out before we even go into court?’ And that's all about [parents’] feedback to us, which is not the typical way they [court hearings] go.”*
Alleviating stigma	*“I've told parents plenty of times ‘look, I don't think you're the worst mom in the world because your kid got arrested. Your kid is old enough to know right from wrong… Everybody makes mistakes, and I'm sorry that you have to be here, but we're not just thinking you're a terrible parent because your kid got arrested.’”*
Independent living	*“Because my girls tend to be older clientele, because they're 16, 17, sometimes 18 years old. If I see that the parent engagement or lack thereof is standing in the way of my client's ability to kinda break the cycle for themselves and their child, I a lot of times will try to help them get into independent living, and maybe even get them out of the current household and get them on their own to see if we can make more progress with them out of that environment where the older generation is refusing to recognize that there's a need for change. But that is specific to my clients.”*
Court action	*“We really can't make them participate all that much. I can request that they show up. If it's a court hearing, they absolutely have to show up, but other than that, they're not required to show up to office visits. It's not in the rules of probation that they do. If we just have no contact from parent or child, then we would take them to court for compliance, but that's pretty much all that we can do. We can also take the parents to court for compliance if it's just like, parents don't take them there, and they're 12. They can't get places on their own. We can still take it to court and tell the judges that it was primarily because the parent was not helping their child.”* *“I feel like the parents who want nothing to do with it I haven't been able to find a good strategy to really get them into it besides like having to tell them, ‘Hey, if you don't get them to their services, then we're gonna have to go to court.’ Which I don't like to use that… I don't like have to more or less threaten them.”* *“Ultimately, they [parents] can be incarcerated for that [not engaging]. That would be the extreme if a parent just really is not engaging. But at the same time, if a parent's gonna dig their heels in and be like, ‘This is not my problem. This is my kid's problem. He's the one that got himself here. He needs to get himself out’ … We can try as much therapy as we can try, but if they're not open to changing … It's just like someone who has a substance abuse problem. If they don't recognize that there's a problem, we can't convince them that there is until they're ready to recognize that for themselves.”*

##### 2.4.3.3 COVID-19

Data collection began prior to the COVID-19 pandemic and continued through the pandemic. Thirty-three dyads were recruited before the pandemic and 44 dyads were recruited during the pandemic. The only correlation between the COVID-19 pandemic and any measured variables was an increase in household income after the pandemic began.

## 3 Results

### 3.1 Demographics

#### 3.1.1 Legal staff

Legal staff were primarily female (60%; *n* = 9), white (93.3%; *n* = 14), non-Hispanic (93.3%; *n* = 14), and between the ages of 26–35 (40%; *n* = 6). All interview participants had been in their current position for at least a year, had at least a bachelor's degree, and reported high job satisfaction (Table 4).

**Table 4:** Legal staff demographics*.*

Demographics	*N*	Percent
Gender (*N*, % female)	9	60.0
Race (*N*, % white)	14	93.3
Ethnicity (*N*, % Non-Hispanic/Latino)	14	93.3
Age (*N*, % between 26 and 35)	6	40.0
Length of time in current position (*N*, % less than 1 year)	0	0.0
Education (*N*, % with at least a bachelor's degree)	15	100.0
Job satisfaction (*N*, % at least satisfied)	15	100.0
Total	15	100.0

#### 3.1.2 Caregivers

Caregiver age ranged from 20 to 68 years with most caregivers being between age 41–50 (40%, *n* = 31). Caregivers were predominantly female (87%; *n* = 67), White (75%; *n* = 58), and non-Hispanic/non-Latino (86%; *n* = 66). Caregiver's relationships to their adolescent was most often mother (77%, *n* = 59), with other relationships including father, grandparent, other relative, and non-relative. Thirty-nine percent of caregivers were married (*n* = 30), 18% of caregivers were in a committed relationship (*n* = 14), and 43% were single (*n* = 33). Out of caregivers in a relationship, the majority (52%, *n* = 40) lived in the same home as their partner. Seventeen percent of households included a grandparent. The majority of households spoke English as their primary language (95%, *n* = 73). Over half of respondents (*n* = 42) had a household income of less than $39,000 per year with 23% (*n* = 18) reporting an income of less than $20,000 per year. Twelve percent of caregivers did not own a vehicle (*n* = 9) and 43% of caregivers only had one vehicle for the household (*n* = 33). Sixteen percent of caregivers scored positive for problematic alcohol use (*n* = 12) and 21% acknowledged illicit substance use (*n* = 16).

##### 3.1.2.1 Life stressors

Caregivers reported high overall levels of life stressors with a mean of 4.6 (SD = 3.1) stressful life events occuring in the last year. There was a wide range of scores with some caregivers acknowledging no life stressors and others endorsing a maximum of 15. Most caregivers identified between 2 and 7 stressful events in the last year. Thirty percent (*n* = 23) of caregivers had moved or relocated in the last year. Twenty-one percent (*n* = 16) of caregivers had separated from their youth's other caregiver in the last year while 9% (*n* = 7) had reconciled with their youth's other caregiver. Thirty-five percent (*n* = 27) of caregivers had experienced the death of a close family member and 23% (*n* = 18) had experienced the death of an important non-family member.Thirty-six percent (*n* = 28) of caregivers reported extended family conflict and 18% (*n* = 14) of caregivers reported experiencing their own legal problems. Twenty-five percent (*n* = 19) of caregivers reported being separated from their youth during the last year and 69% (*n* = 53) reported that their youth had problems at school. Twenty percent (*n* = 15) of caregivers reported that their child had an accident or injury in the last year, 20% (*n* = 15) reported that their youth had medical problems, and 36% (*n* = 28) reported that other close family members had medical problems. Forty-seven percent (*n* = 37) of caregivers reported having financial problems in the last year. Twenty-one percent (*n* = 16) of caregivers reported having work problems in the last year and 18% of stated they had lost their job in the last year. Hope and life satisfaction were low among caregivers, with mean scores of 10.39 (SD = 2.96) and 10.17 (SD = 2.90) respectively. Caregivers also reported high levels of family conflict (*M* = 2.72, SD = 1.24), low levels of child monitoring (*M* = 31.66, SD = 4.98), and low levels of support for their children (*M* = 2.90, SD = 0.65). Full quantitative results are listed in Tables 5, 6.

**Table 5:** Individual level variables from caregiver surveys*.*

Variables	*N*	Percent
Age (*N*, % between 41 and 50)	31	40.3
Gender (*N*, % female)	67	13
Race (*N*, % white)	58	75.3
Ethnicity (*N*, % Non-Hispanic/Non-Latino)	66	85.7
Relationship to Teen (*N*, % mother)	59	76.6
Relationship status (*N*, % single)	33	42.9
Household composition (*N*, % partner resides in household)	40	51.9
Primary language(s) spoken at home (*N*, % English)	73	94.8
Number of vehicles in household (*N*, % two or more)	35	45.5
Household income (*N*, % $20,000–$39,000)	24	31.2
Caregiver substance use		
Drug use	16	20.8
Problematic alcohol use	12	15.6
Total	77	100

**Table 6:** Measure means and standard deviations from caregiver surveys*.*

Measures	*M*	SD
Caregiver support	2.90	0.65
Caregiver monitoring	31.66	4.98
Life stressors	4.61	3.13
Caregiver hope	10.39	2.96
Caregiver life satisfaction	10.17	2.90
Family conflict	2.72	1.23

### 3.2 Legal staff opinions on caregiver engagement

#### 3.2.1 Defining caregiver engagement

Many youth legal staff defined caregiver engagement as caregivers attending appointments with their youth and assisting their youth with transportation to their probation requirements. These requirements included drug screens, community service, and therapy. Some staff expanded this definition to include caregiver involvement in all areas of their child's academic, social, behavioral, and emotional wellbeing. One staff member defined engaged caregivers as those who “*participate in the decision-making*” and have “*a voice.*” Another staff member defined engaged caregivers as those who are actively “*seeking out questions from the service providers like, ‘What can we be doing at home to follow up on this stuff?’*”.

#### 3.2.2 Impact of caregiver engagement

Many officers described an increase in probation requirement completion when youths' caregivers are involved. One staff member stated, “*If you don’t have that parent pushing their child to go and get their community service done, to do their programming, stuff like that, I see that often result in them going up to court because they are not getting their requirements done in time.*” Some staff stated that youth take the requirements more seriously when their caregivers are involved, and others attributed the improvement to increased encouragement from caregivers. One staff member noted. “*…having the parents involved in the process… can be encouraging to the kids so they see that their parents care and they want to help them.*” Several staff members talked about the added accountability that caregiver engagement brings, with one staff member adding, “*Even if the juvenile doesn’t make those changes [in behavior] as long as we don’t have parents enabling, the juvenile at least gets the clear consistent message that these types of behaviors are gonna have consequences.*”

#### 3.2.3 Current state of caregiver engagement

There was a range of responses regarding current levels of caregiver engagement at the youth legal centers. While there was diversity in perspectives, many legal staff described an overwhelming lack of caregiver involvement in the caregivers they interact with. One of these staff members stated, “*Unfortunately, the majority of what we see is the kid gets themselves to all their appointments and we see the parents at court one every three months.*” Most often, legal staff described dichotomous groups wherein some caregivers were “*extremely engaged and helping them [youth]*” and others who “*are just like, ‘well this is on them [youth]. They were the ones that got themselves in trouble. They need to do all this.’*” Some staff acknowledged that caregivers could become engaged depending on how they were approached, and one staff member stated that the majority of the caregivers on their caseload can become positively engaged if they feel that the system is on their side:

“Oh the majority of them [parents] are [positively engaged]. Especially when you get to the end of the whole thing. They may not start out that way, but usually you can win them over… I'd say most parents are pretty aware of what it is their kids are and aren't doing, they just feel the need to defend them in the moment from the big bad legal system. They'll figure out the systems really on their side, we usually to see much better results.”

### 3.3 Barriers to caregiver engagement

#### 3.3.1 Life stressors

Many legal staff members spoke about life stressors as a barrier to caregiver involvement. Financial and work stress were most commonly mentioned and included both the fines and fees of probation as well as time missed from work in order to help youth attend probation requirements. One staff member said “*usually the first thing they [parents] want to know is ‘how much is it going to cost me? Am I going to have to take off work.’*”

#### 3.3.2 Transportation and treatment distance

Many staff members talked about the difficulties for caregivers to get teens to and from outpatient appointments. One staff member said “*We’re a rural community so for someone in [town name], that’s 30 min for them to get in and some parents can’t do that.*” Other staff members spoke to the challenge of involving caregivers in inpatient treatment. A lack of local residential treatment meant that teens had to “*travel pretty far away, which makes it difficult for a parent to be able to go and participate and see them.*”

#### 3.3.3 Caregiver outlook

Some staff members talked about resistance in caregivers due to perceived judgement from the legal staff. “*We do have a lot of parents who… they’re definitely showing their attitude when they first get here. I think its because they think we’re thinking they’re just bad parents,*” voiced one staff member. Poor outlook was reflected heavily in caregiver surveys as well.

#### 3.3.4 Substance Use

A common concern among juvenile justice staff was caregiver substance use. One staff member stated “*Some of the parents use with their kids… they don’t see the difference or they don’t recognize it.*” Another staff member suggested maintaining different approaches towards caregivers who use substances and those who don't.

“*there are families we see where the parents are not drug users and the children have become drug users in some way. And that is a very different dynamic than the families where the parents are drug users and the kids are drug users. And so their approach to that has been no different. I think you've got to approach those situations in a different way. I don't know that you can treat those circumstances the same and expect to get great outcomes by just treating the children.*”

#### 3.3.5 Lack of information

Multiple staff members were concerned that caregivers were not properly informed about the purpose of legal requirements or their rights in the process:

“I think that the reason kids and families struggle meeting the requirements is that they don't always understand the purpose of them.Then people feel like, ‘well, I'm just getting all these requirements and it’s just really cookie cutter. Everybody has to do everything…’ No one’s asking questions, or no one’s really talking about why is that specific requirement or referral necessary.”

Another staff member added:

“I think we miss that informed piece at the beginning, where parents aren't informed that they do have a voice. And so, we'll ask questions to engage them, but unless you're empowered and believe that your voice can and will be heard, even if it’s not agreed with, I think that’s an engagement piece that we lack.”

#### 3.3.6 Lack of responsibility

A common theme among legal staff was a perceived lack of ownership by caregivers. “*They don’t take ownership in it being a family issue. They see it as just the kid’s issue and not a family issue*,” stated one staff member. In addition, staff members brought up a lack of perceived need for intervention from caregivers. “*Quite often [parents] are in denial or they’re minimizing how much of a problem it is. They’re also very defensive about their child’s drug use. ‘It’s not as bad, they just got caught,’ those kinds of…* ‘*Their friends are doing it, they didn’t get in trouble so why is my kid in trouble?*’” Staff members also described confusion among caregivers regarding the overlapping authoritative role that they now shared with the youth legal system “*we have kids who come in who get arrested and as soon as they get home, their parents are punishing them, giving them consequences, knowing ‘yeah, he could still get put on probation, but he’s still my kid,’ and then we have other parents who are like ‘we wanted to see what you guys did first.*’”

#### 3.3.7 Lack of parenting skills

Several staff members discussed a lack of skills as a barrier for caregivers, “*certain parents have done the best that they can do, and the child’s needs may exceed that ability,*” stated one staff member. Another staff member added “*he [youth] and his mom, I know that they care about eachother, but they just don’t know how to keep situations from just blowing up. They’re just not good at it, everything turns into an argument.*”

### 3.4 Staff strategies for caregiver engagement

#### 3.4.1 Respect for caregiver's schedule

Scheduling conflicts was a common barrier identified by youth legal staff and some staff members identified that they have implemented strategies to help overcome this. One staff member attempted to include other family members in appointments if the primary caregiver was not available to attend the appointment with the youth. Another described their efforts to work around caregivers' schedules “*Friday afternoons are not ideal on my schedule because that’s when I’m trying to finish last minute work, but I’ve got a kid whose mom works every day but Fridays so we always schedule her on Fridays.*” The staff member added that they are prompt with each of their appointments so that the caregiver knows their time is valued.

#### 3.4.2 Frequent communication

There was a range of responses about communication with caregivers. Some staff members indicated that they rarely call caregivers, while others identified this as a commonly employed strategy. One staff member said “*If worse comes to worst and [the caregiver] can’t make it, I’m definitely calling and talking to them and making sure that what the juvenile is reporting is the same as how they feel. Making them aware of failed drug screens and making them aware of problems at school that they might not know about.*” Another staff member seconded this strategy “*What I tell the parents is…if you can’t be here and your kid has to walk over after school, call me and let me know how things are going so we can kind of maybe check in over the phone and try to keep them engaged in that.*”

#### 3.4.3 Psychoeducation

Some legal staff indicated that they work to educate caregivers on the topic of parenting skills as a means of addressing this barrier. Some staff advised caregivers to be more supportive and less harsh with their youth to encourage them to be honest about substance use and other issues. One staff member talked about educating caregivers that their youth should pay the fines set by probation and not the caregiver to help the youth take responsibility for their actions. Other staff members described speaking with caregivers about appropriate boundaries for their youth. Some staff advised caregivers on how to engage with their youth as described by one of these staff members: “*I at least send [parents] out with at least verbal tools, verbal praise, and maybe suggestions… some ideas of how to engage with their child.*”

#### 3.4.4 Family oriented programs

A few legal staff stated that new programs that include the families are very helpful but are not widely implemented. One staff member said: “*We do have a few programs that actually include the parents. Those obviously are pretty engaging and actually allow the parents who might not be receptive to kind of being a part of it actually enjoy doing those, but there’s only one or two that we have like that.*” Another staff member said that after receiving feedback from caregivers they implemented a facts panel that is utilized in specific situations which includes multiple members of the youth's family, community, and legal staff in which staff can ask questions of the caregivers and caregivers can ask any questions that they have. This staff member said:

“it’s really making the parent part of the team in that moment to figure out what the recommendation at that emergency detention hearing’s going to be. Also, we do a little exit survey at the end, and parents are like, ‘He’s been arrested many times before, but that is the first time I felt heard. That is the first time I understood what was going to happen in a court hearing.’”

#### 3.4.5 Alleviating stigma

Several staff members described efforts to alleviate stigma and reduce shame among caregivers of legally involved youth. One staff member said:

“I've told parents plenty of times look, ‘I don't think you're the worst mom in the world because your kid got arrested. Your kid is old enough to know right from wrong… Everybody makes mistakes, and I'm sorry that you have to be here, but we're not just thinking you're a terrible parent because your kid got arrested’”

#### 3.4.6 Independent living

One staff member whose caseload consisted only of older youth said that if caregiver engagement is lacking, they will try to get the youth into independent living to identify if they “*can make more progress with them out of that environment where the older generation is refusing to recognize that there’s a need for change.*”

#### 3.4.7 Court action

Some staff members said that they had very few strategies for caregiver engagement besides court action. One staff member said “*We really can't make them participate all that much. I can request that they show up. If it’s a court hearing, they absolutely have to show up, but other than that, they're not required to show up to office visits. It’s not in the rules of probation that they do.*” One staff member said that they do not like having to threaten with court but that they feel they have no other option:

“I feel like the parents who want nothing to do with it, I haven't been able to find a good strategy to really get them into it besides like having to tell them, ‘Hey, if you don't get them to their services, then we're gonna have to go to court.’ Which I don't like to use that… I don't like have to more or less threaten them.”

Another staff member stated that, “*If [parents] don't recognize that there’s a problem, we can't convince them that there is until they're ready to recognize that for themselves.*”

## 4 Discussion

The current study aimed to gain a more comprehensive understanding of caregiver engagement in the youth legal system who are at risk for substance use including barriers to engagement and strategies to increase engagement. These findings can assist personnel by providing a nuanced understanding of the families served in the youth-legal setting, affording opportunities to tailor procedures and interventions to best meet the needs of the youth and families they serve. The current study highlights the challenges to engaging families in the probation process and the simple strategies POs often use to engage them. Interviews with legal staff were paired with quantitative data gathered from caregivers recruited from the same communities to highlight the wide range of challenges experienced by legally involved families of youth at risk for substance use, including, but not limited to, experiences of poverty, ongoing stressful life events (perhaps including the youth's legal involvement), and caregivers' own behavioral health needs.

Most youth legal staff defined caregiver engagement within the context of probation requirement completion by attending appointments and transporting youth. Even with this relatively limited working definition, JPOs most often described caregivers as disengaged. Disengagement was often described as putting an onus on the youth to complete probation requirements with limited or no caregiver support. While JPOs acknowledge diversity in caregiver participation, with some describing families as positively engaged, it was noted that youth with caregivers who did not engage in the process were less likely to meet probation requirements. Not meeting probation requirements can result in serious escalation in a youth's case, including new charges and detainment. Poor caregiver engagement in the probation process could have impactful, cumulative effects on the developmental trajectories of their legally involved children (65).

Challenges to engaging caregivers may not be surprising, especially when considering the context in which these caregivers try to parent. Quantitative data consistently revealed practical factors that could dampen caregivers' ability to fully participate in their child's legal proceedings. Caregivers reported experiencing high levels of poverty, and less than 50% of families in the current study had reliable access to two cars. JPOs were also aware of these barriers, noting financial stress and transportation issues as prominent barriers to engagement. Considering that JPOs often define engagement by appointment attendance and transportation, it could be that caregivers do not have the resources to engage in the probation process as expected. Further, probation requirements may include paying a fine or other financial burdens to families. Even though the youth (not the caregiver) is required to pay any fines, in practice it may not be realistic to expect the youth to be able to generate the income required without the assistance of a caregiver. Barriers to attending treatment are well-documented for families experiencing poverty, with evidence suggesting that practical barriers often contribute to poor treatment engagement more so than negative beliefs about treatment (66).

Findings also suggest that caregivers experience their own behavioral health concerns that may impede their ability to assist their child and their child's JPO during the probation process. Rates of caregiver problematic substance use were high, with 16% scoring positive for problematic alcohol use and 21% endorsing illicit substance use. JPOs also acknowledged caregiver substance use as a significant barrier to treatment engagement. To participate in the study, youth had to endorse problematic substance use themselves, so rates of caregiver substance use may be higher in the current sample than in other samples of families participating in probation programming. However, it is worth noting that youth in the juvenile legal system frequently experience substance use difficulties that could result in further legal involvement (67–70). Unfortunately, findings suggest that youth in the legal system may not have caregivers who can support them in addressing substance use difficulties, especially if they have more permissive views of substance use and ongoing substance use difficulties. Programs designed to connect caregivers with substance use treatment services may be a valuable intervention to decrease recidivism and increase timely completion of probation requirements among youth.

Further, caregivers' experiences were characterized by high stress and low life satisfaction. Stressful experiences are experienced cumulatively, meaning that additional stressors result in further stress response and potential for impairment. The more frequent these events, the more likely functioning will be impacted. Functioning across domains of home, work, relationships, and emotional regulation can deteriorate because of chronic stress (71, 72). Further, it is worth noting that familial legal involvement represents yet another stressful event that caregivers must cope with. Especially given that probation requirements often necessitate caregiver involvement, high stress may be impeding caregivers' ability to be engaged, even if they want to be. Notably, evidence would suggest that caregivers have a generally negative outlook on the future, characterized by hopelessness and poor life satisfaction, which was associated with low caregiver support and monitoring of their child. This is consistent with literature demonstrating that caregivers experiencing the chronic stress of poverty (and other associated stressful experiences and circumstances) are more likely to engage in authoritarian caregiving (e.g., Less responsive, harsh caregiving) (73, 74). Thus, while caregivers' lack of engagement can be frustrating for JPOs and make it more challenging for youth to meet probation requirements, it is worth acknowledging that many caregivers of legally involved youth are likely managing a myriad of other stressful experiences, behavioral health issues, and circumstances and may feel unable to fully engage in yet another stressful experience, especially one they perceive as being outside of their control or not their problem.

In addition to these challenges, caregivers are faced with learning how to navigate the juvenile legal system. JPOs noted that caregivers are often uninformed with the probation process and confused about their overlapping roles with JPOs. JPOs described caregivers as unsure whether to take charge of a situation or defer to probation (i.e., “*Let probation handle it.*”*).* Perhaps this confusion is unsurprising given the founding principles of *parens patriae*. While efforts have been made to involve caregivers in the process, including through family programming mentioned by legal staff participating in the current study, the youth legal system was designed to give courts jurisdiction over youth and dictate their care. Engaging caregivers in the process is often viewed as critical by JPOs. Still, caregivers of legally involved youth commonly report feeling as though they do not have a voice and are receiving punishment alongside their child (75), which was echoed in the current study. Thus, caregiver confusion regarding their role and how they would best interface with probation may be expected, especially if they do not feel they can use their voice and ask questions. Of course, accomplishing these tasks can be made more difficult in the context of ongoing stress and personal behavioral health concerns.

Critically, JPOs varied wildly in their willingness and ability to engage families, and no uniform procedures for family engagement were described. Ultimately, how a family is engaged would depend on the JPO they were assigned to and how that JPO chooses to approach families. Many JPOs noted their attempts to provide parenting guidance to caregivers, namely regarding how caregivers approached and interacted with their youth. Examples of guidance given within the current study broadly aligned with behavioral principles and authoritative parenting styles, which is consistent with evidence-based programs for addressing problem behavior in youth (76, 77). Few JPOs identified interpersonal strategies for increasing engagement. While some noted the importance of alleviating the stigma and shame that may come with their youth's legal involvement, most identified administrative and logistical approaches to foster caregiver engagement. Simple strategies that JPOs used included deferring to caregivers' schedules for appointments and frequently communicating with caregivers about their child's case. Yet other strategies noted by JPOs included system-level actions to dictate caregiver behavior, including moving youth into independent living or further court action.

These findings build upon prior literature that examined legal staff interactions with caregivers of legally involved youth. The opinions expressed by legal staff in this study are largely consistent with views and interventions identified in prior studies (20, 22) as previously discussed. The present study contextualizes these findings by including survey data from caregivers to better understand the factors impacting caregiver engagement.

## 5 Limitations

Several limitations of this study should be noted. In order to achieve an adequate sample size, data from two counties were included in the study. While no county specific associations were identified during data analysis, it is possible that the inclusion of data from two separate counties could have impacted the results. Further studies should be completed to corroborate the findings from the present study. Both counties involved in the study are majority white counties leading to minimal racial diversity in the sample population. Quantitative measures were not stratified by demographic factors in this study due to a limited sample size. However, it is anticipated that these factors may play a role in caregiver engagement. Further studies are necessary to identify the specific impact of demographic factors such as race, gender, and age on caregiver engagement. Additionally, only youth at risk for problematic substance use and their caregivers were included in the sample population for survey data. As this is a subset of all legally involved youth, we do not know the degree to which the quantitative characterizations identified in this study generalize to legally involved youth and their caregivers nationwide. Youth who were wards of the state or currently detained were not eligible for the study. Additionally, data was not collected regarding families' prior involvement with child protective services. This is an important limitation to note as caregivers' relationship with the legal system and their youth may change when their youth are actively detained, when youth are temporarily removed from their care, or after termination of parental rights. Furthermore, caregiver's own prior involvement with the legal system was not documented. This is an important limitation as many youth who are involved in the youth legal system also have caregivers who have been involved in the legal system. It is presumed that a caregiver's own personal history with the legal system may impact their ability to engage with legal services on behalf of their youth. Additional studies are necessary to explore the impact of prior involvement with child protective services and the legal system on caregiver engagement. Data regarding the presence of life stressors was collected in both the caregiver surveys and the interviews with legal staff. However, it is possible that not all life stressors were captured with the questions asked including but not limited to physical disabilities that a caregiver may have. It is important to expand on research in future studies to identify additional life stressors that may be present. Additionally, caregivers with more time and resources available may be overrepresented in this study due to the requirement that dyads had to be reachable by phone and participation in this study was voluntary. Because this study sought to include caregivers of all youth at risk for substance use who encounter the legal system including those who undergo formal processing and those who do not, it did not include the perspectives of legal counsel such as defense or prosecution. Exploring these perspectives in further studies may be able to provide additional insight into caregiver engagement with the youth legal system. This study does not include qualitative interviews with caregivers. Further exploration into the caregiver perspective is needed to fully characterize caregivers of legally involved youth. This study provided descriptive statistics to support qualitative descriptions of caregivers and caregiver engagement. More research is needed to understand moderators and mediators of caregiver engagement in their youth's legal services from a balanced perspective that includes caregiver strengths as well as caregiver challenges. Lastly, the youth perspective is important to shed light on the impact that caregiver characteristics have on their legally involved youth and their experience with the legal system. Since children may look to their caregivers to model characteristics such as hope and expectations for the future, it is important to understand with future studies if there is a relationship between these youth characteristics and caregiver characteristics.

## Data Availability

The datasets presented in this article are not readily available because of state court restrictions surrounding the multiple vulnerabilities of the participant population. Requests to access the datasets should be directed to Annie Turner, pcovington184@marian.edu.
